# Understanding Cancer Care for Nursing Home Residents Living With Dementia: An Ethnographic Study

**DOI:** 10.1002/pon.70184

**Published:** 2025-05-31

**Authors:** Olivia Claire Robinson, Claire Surr, Laura Ashley

**Affiliations:** ^1^ School of Humanities and Social Sciences Leeds Beckett University Leeds UK; ^2^ Faculty of Medicine and Health School of Medicine Leeds Institute of Health Sciences University of Leeds Leeds UK; ^3^ Centre for Dementia Research Leeds Beckett University Leeds UK

**Keywords:** cancer, care needs, dementia, nursing homes, older adults, oncology, primary care, qualitative

## Abstract

**Objective:**

In the United Kingdom (UK), 1 in 13 people living with dementia also have cancer. At some point, 41.3% of this population group will require care home support. Limited research has examined the care and support needs of people with dementia and comorbid cancer (DCC) in nursing homes (NHs). This study aimed to explore the care experiences of NH residents with DCC, their families, nursing home staff (NHS) and healthcare professionals (HCPs), and to identify challenges and good practices, to develop recommendations for practice improvement.

**Methods:**

A focussed ethnography using interviews, observations, informal conversations, and review of care plan documentation. Data were analysed using ethnographically informed reflexive thematic analysis.

**Results:**

Eight HCPs, Six NHS, 5 family caregivers and 7 residents with DCC were recruited from five NHs in Northern England. Two themes were developed: *Complexities around cancer referral and treatment decision‐making* and *Relative invisibility of a resident's clinical cancer diagnosis*. Findings suggested residents with DCC were not included in best interest decision‐making due to the potential distress knowing about a cancer diagnosis would cause. Families, HCPs and NH staff made collective decisions on the behalf of residents. Often cancer referral was deemed not appropriate. Thus, people with dementia had a clinical‐only cancer diagnosis, resulting in limited formal information about the cancer in care documentation and staff knowledge. Potential consequences of having a clinical‐only cancer diagnosis included: misattributing cancer symptoms to dementia, reactive care responses to cancer symptoms and the possibility of inadequately managed cancer symptoms.

**Conclusions:**

Implementing earlier discussions about feasible care outcomes is crucial. These conversations should include considerations around hospital referral for oncology care or care through palliation in the NH. Without appropriate recognition of a clinical‐only cancer diagnosis and support for staff it could lead to advancement of symptoms that might be challenging and less well managed. We outline several recommendations to support NHS to deliver person‐centred care to residents with DCC.

## 1 Background

In the UK, 1 in 13 people living with dementia also have a cancer diagnosis [1]. Research indicates people with DCC have worse outcomes than those with cancer alone, including being more likely to be undiagnosed, experience emergency hospital admissions, and receive fewer oncology referrals [2, 3, 4] People with DCC also have complex care needs (e.g., pain), and often, their ability to communicate these needs can be impacted due to the severity of their dementia [5, 6]. This results in people with DCC receiving less pain relief [7], potentially leading to an increased presence of symptoms perceived to be neuropsychiatric (i.e., agitation), but which are related to under‐treated cancer pain [8]. At some point, 41.3% of those people with dementia and comorbid cancer (DCC) will require care home support [1]. However, limited research has examined the care and support needs of this group. The research that has been conducted has focussed predominantly on pain in residents with DCC, highlighting under‐identification and poor management of pain in this group [7, 9]. Studies have also explored the presence of symptoms such as agitation, distress, and sleep disturbances, which are often attributed to dementia but can result from poorly managed cancer pain. Consequences of poor cancer symptom management, including pain, can include acute cancer episodes (e.g., bowel obstructions), increased emergency department admissions and hospitalisations, and poor quality of life for the person with DCC [2, 3, 4, 8].

Dementia may complicate clinical decision‐making about cancer referrals, for example, by raising questions about the appropriateness and value of cancer investigations. To our knowledge, there are no guidelines to support and inform cancer‐related decision‐making for people living with dementia [10, 11, 12]. No research to date has explored the care receipt and delivery experiences for this somewhat hidden group. This study, therefore, aimed to explore the care experiences of nursing home (NH) residents with DCC, their families, NH staff, and healthcare professionals (HCPs). Specifically, the current research explores current care practices, associated challenges, and identification of strategies to provide good quality person‐centred care (PCC).

## 2 Methods

### 2.1 Design

A focussed ethnography [13] involving participant observations, semi‐structured interviews, review of care plan documentation and informal conversations. This method enabled the inclusion of people with moderate to severe dementia that were unable to take part in interviews [14], facilitated the recording of care plan documentation, and provided valuable insights into care practices that were used to inform participant interviews.

### 2.2 Participants and Recruitment

Participants were recruited from five NHs in Northern England. NHs were independently contacted by the first author or identified and recruited via HCPs from local palliative care teams who supported people with DCC in NHs. These HCPs approached NH managers with an organisational information sheet. If the NH manager agreed in principle to being contacted about the study, the HCP gave the NH name and contact details to the first author. Prior to consent, the first author contacted the NH manager to provide further information about the study. The NHs were from different urban locations in Northern England, privately owned and ranged from 20 to 30 bed NHs to larger, 40–50 bed NHs.

Purposive sampling [15] was used to recruit NH residents with DCC and other participants with varied roles and experiences of providing care for this group. This included NH staff, families, and HCPs. People with dementia were eligible to take part if they (1) had a diagnosis of dementia, and a current or recent clinical (a suspected diagnosis based on symptoms, history, and examination, without definitive tests) or confirmed (a diagnosis verified through objective tests like labs, imaging, or pathology) diagnosis of cancer; (2) had received or was receiving care for their cancer and/or symptoms and side‐effects and (3) lived within a participating NH for at least 1‐month. With assistance from nursing staff, participant eligibility was discussed with the first author and potential participants were identified and approached. NH staff and HCPs were eligible if they had recent experience (within the last year) caring for a NH resident with DCC and were aware of the cancer diagnosis. Families needed to have cared or were currently caring for someone with DCC and visit regularly (once per month). Sample size was determined based on previous ethnographic studies conducted in care homes [16, 17]. We aimed to recruit up to 20 people to ensure diversity in key participant characteristics and achieve sufficient data saturation to address the research questions.

### 2.3 Informed Consent

Written consent was obtained for interviews with families, NH staff and HCPs, access to care plan documentation and individual observations (with residents). If residents did not have the capacity to consent to the research, a personal or nominated consultee was approached in line with the Mental Capacity Act (2005), to provide advice on their wishes. Assent to take part was assessed on an ongoing basis by the first author and NH staff who knew the resident during data collection (e.g., to ensure the participant did not express unwillingness to take part or show signs of distress) [18]. General observations of day‐to‐day practices conducted in communal areas (e.g., lounge, dining areas) did not require written consent as all observational data was anonymised at the point of collection.

### 2.4 Ethnographic Data Collection

Data were collected between March 2019 and January 2020 across five NHs. Data collection was undertaken by the first author for their doctoral thesis, prior to which they had masters level experience of qualitative applied health research, including with people living with dementia in care homes. There were four methods of data collection used.

- General observations of the routine patterns and behaviours of care within the NHs;
- Individual observations of participating residents and accompanying informal conversations;
- In‐depth interviews with family/friends, NH staff and other HCPs involved in the provision of care to recruited residents;
- Extraction of data from care plan documentation (including demographic data) to provide insight into a resident's care.

Observations took place within NH communal areas, staff rooms and resident bedrooms. No private or personal care was observed (i.e., washing or dressing). An observational framework was used to guide observations (see Supplementary File 1). Observations and informal conversations were recorded in handwritten field‐note diaries and typed up and expanded into more detailed notes on the same day to aid recall.

Interview topic guides for participant groups were developed by the research team in line with existing care guidelines [19] and previous literature, in collaboration with the Lay Advisory group (LAG). Topic guides were flexible to ensure they were personalised for each participant, with specific questions tailored to understanding individual situations, relationships between participants and events that had been observed, and the meaning behind those events. Interviews were conducted in designated rooms on‐site at each NH, audio‐recorded and transcribed verbatim. All participants were given ID numbers to anonymise the transcripts.

### 2.5 Lay Advisory Group (LAG)

The LAG supported all aspects of the studies design and delivery and included two caregivers for people with DCC and three academics with experience in DCC research. The LAG met every 6‐months to discuss study design, delivery methods and provided feedback on study documents and initial themes and sub‐themes.

### 2.6 Ethical Approval

Ethical approval was obtained by the National Health Service Research Ethics Committee in Yorkshire and & the Humber—Leeds Bradford (18/YH/0278).

### 2.7 Data Analysis

Data analysis was an iterative process, conducted by the first author, which began at the start of data collection, with early analysis being used to inform and hone subsequent data collection, focussing on exploring the content, the patterns, and key events in the data. Data was analysed using reflexive thematic analysis [20]. Reflexive thematic analysis allows for an *iterative and evolving coding process*, which suits the *exploratory nature of focussed ethnography* [20]. All data (interview transcripts, observational notes and reflections, and care plan documentation notes) were read and re‐read, and reflections of initial thoughts and observations were captured in the page margins by the first author. Codes were developed to help describe and classify the data with reference to the research questions (i.e. a sentence may be labelled ‘cancer‐referral decision‐making’ and ‘level of cancer‐related care knowledge’). The codes continued to be discussed and refined with the LAG and wider research team as further data was analysed. On completion of the coding, definitive themes were finalised through review and discussion. Each theme was described in detail, with supporting evidence (i.e. quotes, fieldnotes) reviewed. Themes were refined iteratively until consensus was reached, and anonymised quotes illustrated and validated the findings. All data were analysed using NVivo 11pro.

### 2.8 Reflexivity

Reflexivity was essential in acknowledging the first authors positionality. The first author had no direct experience working in a care home, their perspective was shaped by research expertise in applied health research rather than a clinical or staff role. Reflexivity was used to reflect on any judgements and preconceptions the first author had, to facilitate understanding of potential biases, and to identify the subjective observations which supported the research questions [21]. Reflections were documented in a reflexive journal, and informed ongoing data collection and the identification and development of themes and sub‐themes, while helping to minimise researcher biases.

## 3 Results

### 3.1 Participants and Data Collection

A total of 19 interviews were conducted (6 NH staff, 8 HCPs and 5 family caregivers), lasting between 30‐min to one‐hour. The observations of care experiences of seven residents living with DCC (approx. 90 h over 10 months), and broader NH observations (approx. 170‐h over 10‐month) were also documented. This included the recording of informal conversations and the review of care plan documentation for the seven residents with DCC (see Table 1 for participant characteristics).

**TABLE 1:** Participant numbers and characteristics (*N* = 26).

Participant characteristics *n* (%)	
Residents living with dementia and comorbid cancer (=7)	
Male/Female	5 (71)/2 (29)
Mean age (years)	84 (range 75–91)
Ethnicity	
White british	6 (86)
Other white background	1 (14)
Type of dementia	
Vascular dementia	3 (43)
Mixed (Alzheimer's and vascular dementia)	2 (29)
Dementia with lewy bodies	1 (14)
Alzheimer's disease	1 (14)
Clinical‐only Cancer diagnoses	
Bowel	1 (14)
Ovarian	1 (14)
Prostate	1 (14)
Skin	1 (14)
Lung	1 (14)
Bladder	1 (14)
Rectal^a^	1 (14)
Healthcare professionals (n = 14)	
Male/Female	1 (7)/13 (93)
Staff role	
Nurse	3 (21)
Healthcare assistant	4 (29)
Nursing home manager	2 (14)
Palliative care specialist nurse	3 (21)
Advanced nurse practitioner (ANP)	2 (14)
Family caregivers (n = 5)	
Male/Female	2 (40)/3 (60)
Relationship to resident	
Child	2 (40)
Nephew	1 (20)
Niece	1 (20)
Spouse	1 (20)

^a^ One participant had a confirmed diagnosis of rectal cancer after further investigations at hospital.

This paper presents two themes that provide an overview of current care practices and the challenges and support needs of NH residents with DCC. Firstly, *complexities of shared cancer referral decision‐making for residents living with dementia* explores decision‐making capacity and understanding the resident's best interests and practical obstacles to accessing oncology services. Secondly, *relative invisibility of a resident's clinical cancer diagnosis* explores prioritisation of dementia‐focussed needs driving practice and consequences related to dementia‐oriented care.

### 3.2 Complexities of Shared Cancer Referral Decision‐Making for Residents Living With Dementia

Most participants with DCC were unable to verbally provide insight into their own care decisions due to cognitive and communication difficulties. For those who did have capacity, families and staff members voiced concern that informing the resident about a potential cancer diagnosis may be too distressing, especially if the information needed to be repeated multiple times to facilitate their inclusion in decision‐making. Thus, decisions often had to be made collectively in the best interests of residents by families, supported by the NH manager and HCPs. However, this was complex, and families often held conflicting views on what might be the best option for the resident. In some cases, HCPs believed families wanted investigations and treatments that might not be in the resident's best interests:

Families can feel obliged to make these decisions rather than being guided towards what might be best for our residents. That’s where things can get sticky because some families want treatment at all costs and that’s about their agenda rather than the agenda of our resident.

(End‐of‐life care co‐ordinator, NH5)

In other cases, NH staff felt residents were ‘*written off’* by HCPs because of the stigma related to age and dementia. This meant people who may benefit from referral and specialist input did not receive this:

NH manager suggested when an individual [referring to resident] has dementia they are written off in the view of hospitals and medical professionals. She felt that they [referring to GPs] should focus on doing what’s right for the person regardless of diagnosis.

(Fieldnotes, NH3)

Families were therefore placed at the centre of these complex decisions and looked to those they saw as experts to help them make decisions. For example, families primarily sought the advice and support from General Practitioners (GPs). There was consensus that the views and expertise of the GP may offer reassurance to families that they were making the right decisions, particularly when this was not to refer.

The GP virtually said the same thing to me, that they wouldn’t recommend him going to hospital…the approach will just be palliative.

(FC (Family Caregiver)3, NH2)

In making these recommendations, a delicate balance had to be struck by HCPs in considering potential benefits a confirmed cancer diagnosis might bring against the potential risks, burdens or harm a resident may be exposed to during investigations. For example, whether treatments would be feasible for the person with DCC to manage/comply with and whether life prolonging interventions were appropriate.

It can be difficult to manage expectations from relatives … I hate to use the term unrealistic but that’s how it feels sometimes that nobody wants to accept their loved one is dying… it’s not always in that person’s best interest to be kept alive for as long as possible. Sometimes it’s in their best interest to stop actively treating them and treating them symptomatically and let them die peacefully.

(Staff interview 14, Advanced nurse practitioner)

NH staff were often left out of discussions and decision‐making, even though they often knew residents well and would be the ones responsible for the ongoing care of the person. Thus, decisions around referral for cancer diagnosis and potential treatment, even if palliative, were complex and required the input of families, NH staff and HCPs. The perspective of the person with dementia was largely not present in this decision‐making process.

To add to the decision‐making complexity, there were well‐being and physical barriers to consider in determining whether referral for cancer investigations was in the resident's best interests. Well‐being barriers included concerns families and staff held about the potential for causing emotional distress as a result of disclosing to the resident the reason for attending an oncology service. Additionally, this could have detrimental impacts due to leaving a familiar environment and the stress associated with attending a hospital appointment.

I wouldn’t want to tell them or upset them, they are happy, our residents are receiving palliative care. We want to keep them until the end. We can cater to their nursing needs”.

(Fieldnotes, NH3)

They won’t know what’s happening to them. It’s scary for them.

(Nurse, NH2)

On reflection [R003] she has declined rapidly, so she would have been too distressed with her hallucinations to attend those appointments.

(Fieldnotes, R003)

Physical barriers included uncertainty between NHs and families about who was responsible for taking a resident to hospital, concerns about transport to get there, and a general sense that attending hospital was a logistical ‘*nightmare’ (nurse, NH3)*. Both families and staff felt hospital staff were unprepared to understand the needs of residents living with dementia and their unique ways of communicating. This could cause distress that might not be easy to support:

The medical side, that was all down to me usually, taking him for the check‐ups then taking him for the operations as well. …I wonder what happens to people who haven’t got anyone to take them to their treatments.

(FC3, NH2)

It can be really difficult, there have been days were because of what’s been going on within the house we’ve been unable to get people, and we’ve had to cancel and re‐arrange appointments because it’s been impossible, if we are short [of staff] already.

(Staff interview 2, Nurse, NH2)

At every stage when we had to go do bloods and stuff, I had to explain that he’s not obnoxious, he’s got dementia. He might lash out and swear at you, he didn’t most of his life, but this is dementia

(FC4, NH5)

All participants suggested the combination of the perceived challenges to oncology referral had to be weighed against the benefit of a confirmed cancer diagnosis (i.e., access to specialist oncology support). Ultimately, participants suggested it was usually, on balance, more appropriate to not refer for most NH residents, given the complexity of their needs.

### 3.3 Relative invisibility of a Resident's Clinical Cancer Diagnosis

While the findings presented have indicated a confirmed cancer diagnosis may not be appropriate and, in many cases, potentially more harmful for the individual, a ‘clinical diagnosis’ unconfirmed via medical tests also presented potential barriers to good care. Findings indicated that a clinical diagnosis meant *limited formal information about the cancer in care documentation* and a lack of staff knowledge, in comparison with their knowledge and understanding of the resident's dementia. There was a *prioritisation of dementia‐focussed needs* driving practice, resulting in the risk of cancer symptoms and needs being overlooked. Consequences of this included *a risk of misattributing cancer symptoms to dementia, reactive care responses to cancer symptoms* and *the possibility of inadequately managed cancer symptoms.*

It was clear from reviewing some care plans that information related to a resident's dementia and implications for care delivery was clearly recorded. However, the same plans contained relatively little information about a resident's cancer. For one resident, despite having a clinical‐only diagnosed bladder cancer diagnosis, this information and its implications were not recorded in sections related to incontinence or pain. However, other medical conditions such as diabetes and asthma were accurately documented:

R007’s care plan has detailed sections dedicated to the resident’s special care needs. These included descriptions surrounding:

**Communication** NH staff describe R007’s current level of ability to communicate and the deterioration due to their dementia.

**Breathing** NH staff outline R007’s asthma and the difficulties they have breathing.

**Eating** NH staff outline what R007’s can/can’t eat due to their diabetes.

**Continence** NH staff outline R007’s incontinence level requires pads as fully incontinent.

(R007 care plan fieldnotes)

For other residents, there was information in daily records of contact with HCPs, but this had not been transferred into the overall care planning document.

27/4 NH liaison assessment ‐ “Reason for the referral—diagnosis of vascular dementia may have developed stomach cancer not clarified by GP”.

1/8 NH liaison assessment

11/3 Doctor visited the home.

“Carers noticed tissue with blood from down below, no complaints of abdominal pain, got urine sample with blood in”.

12/9 “Ovarian cancer for urgent USS at BMI”

(R003 Care Plan Fieldnotes)

Where oncology services had been accessed, a recording of cancer in the care plan was more comprehensive. It was therefore harder for staff to understand, support and manage cancer‐related care needs if they had little or no awareness of the cancer or its severity, and perhaps unsurprising it was not given the same status as dementia when delivering care.

I think dementia is seen more as the active problem because if someone didn’t have dementia then the active problem would be cancer. So, the focus is completely different.

(Nurse, NH4)

I think you could almost say that dementia certainly colours how you see the disease.

(FC3, NH2)

Caregivers and HCPs raised concerns about whether NH staff were able to effectively identify and manage a resident's cancer‐related symptoms, particularly when the type and severity of cancer was unknown, and changes might be attributed to dementia instead.

Dad is changing, his moods are changing. He’s going off his food, he’s tired more. He’s getting more aggressive, there all dementia traits but they are massively out of character for dad even when he’s had wobbly dementia days. If he fiddles with his nose, he can distress himself touching things, that’s got to be a big flag.

(FC5, NH5)

When a resident did not have a confirmed cancer diagnosis, HCPs working in specialist roles believed nursing staff were disempowered to manage and monitor cancer‐related symptoms effectively, due to lack of knowledge about the cancer and variable access to specialist services that have expertise in symptom management:

Well, it's PRN it’s for leg pain and that resident has become immobile and is not sleeping and is crying out and going, mummy and rubbing their legs. The connection between the two isn’t being made

(End‐of‐life care co‐ordinator, NH5)

However, where staff were aware of a cancer diagnosis and this was documented in care records, there were examples of proactive care and management of symptoms by NH staff.

R004 is screaming “arghh” with both hands down his trousers. The nurse says, “I know it hurts”, she places her arm on his shoulder to encourage him to the toilet. When the nurse returns, I ask if the severe UTIs are associated with his cancer, “it is suspected that the UTI and pelvic abscess are all associated with his rectal cancer, but they are unable to continue with an investigation because he struggles to stay still. The most appropriate way to treat R004 is through symptom management.”

(Fieldnotes, R004)

The delivery of cancer care is constantly changing, with residents developing new cancer‐related symptoms that could potentially become more severe. The data emphasised the continuous need for staff and HCPs to monitor a diagnosis (including a clinical diagnosis) of cancer and integrate this information into care plan documentation. This could potentially minimise the risk of emergency presentation of symptoms.

## 4 Discussion

To our knowledge, this is the first qualitative study in the UK to explore cancer‐related care for NH residents with dementia. The findings extend previous insights into identified care disparities for people with DCC [22, 23, 24, 25].

From our overview of care practices, we found low levels of cancer referral for residents living with dementia, who were frail and had complex care needs. We suggest the number of NH residents living with DCC could be higher than reported in current statistics [1] due to the preference to not refer to secondary care. This is consolidated by existing literature that indicates older adults are at an increased risk of experiencing treatment toxicities, 30‐day mortality rates and challenges with compliance with treatment [26, 27].

We found residents with DCC were not included in best interest decision‐making due to the potential distress knowing about a cancer diagnosis would cause. Thus, families, HCPs and NH staff made collective decisions on the behalf of residents. There are no guidelines for cancer investigations or referrals for older adults that are also frail and have comorbidities [10, 11, 12]. This makes decision‐making complex as differing views of families and HCPs can complicate the decision‐making process [28]. Thus, implementing earlier conversations about feasible care outcomes for the resident (e.g., making a hospital referral to receive oncology care or care through palliation in the NH) is crucial and should include the resident (where appropriate), their family, NH manager and HCPs (See Table 2). We identified referral decisions were also impeded by physical and well‐being barriers. This is not an uncommon finding in DCC literature as patient transport to and from hospital, patient and family burden [25, 29] and unmet dementia training needs in hospitals [22, 24, 29] were all identified challenges for people with DCC and their families.

**TABLE 2:** Healthcare service‐level and nursing home practice recommendations.

Recommendations for practice	Suggestions for implementation
Nursing home practice recommendations	
Improve shared decision‐making for residents with residents with dementia and comorbid cancer	
1. If a resident receives a clinical diagnosis, ceilings of care need to be established to identify the feasible outcomes for their care. This conversation should include the resident, where appropriate, their family, nursing home manager and HCPs.	Outline with families the potential cancer care options available (i.e., make a GP referral to receive hospital‐based oncology care or receive cancer care through palliation in the nursing home).
2. If feasible for a resident to be referred to hospital, discussions with caregivers, HCP, residents, and nursing home managers are vital to outline logistics of accessing oncology services.	Outline who will take responsibility of taking the resident, how they will get there, and how many people will be required to go to support the appointment, and follow‐up appointments.
3. If it is in the best interest of the resident not to be referred to hospital, it is important to follow up with external services (i.e., palliative care needs) to identify potential cancer care needs a resident may have.	Identify a point of contact or experienced HCP in specialist palliative care to support the development of an action/care plan.
Improving cancer awareness, documentation, and action planning	
1. A resident's clinical cancer diagnosis and associated symptoms should be integrated into the resident's care plan.	Integrate a residents clinical cancer diagnosis into the ‘health’ section of the care plan, this should also include related needs, and what support has been put in place for the individual. Additionally, the potential implications a clinical cancer diagnosis can have on other aspects of a resident's care should be integrated throughout the care plan.
2. If appropriate, implement strategies to promote assessment and continuous monitoring of resident symptoms (i.e. pain., agitation)	Implement symptom assessment tools into care plan records to support the identification and continuous monitoring of a resident's pain (i.e. abbey pain scale).
3. Ensure written summaries of key information and discussions between all parts of the care triad (HCPS, families, and NH staff) are recorded.	Provide bullet points in care plans of communication with HCP professionals and caregivers and any changes to delivery of palliative care.
Increase nursing home staff training and support to deliver specialist palliative care	
1. Ensure staff have access to external palliative care support services (i.e., contact numbers to local palliative care teams)	Establish a communication pathway from the nursing home to local palliative care teams for seeking their input into care decisions, or for emergency support.
2. Managers to work with local palliative care teams to identify training needs and gaps in knowledge for nursing home staff (i.e., how to support decision‐making, use syringe drivers, symptom management)	Promote specific members of staff to access specialist palliative care training (i.e., macmillan training for nursing home lead care assistant and nurses) to increase knowledge and confidence in symptom management.
	Introduce new training techniques (i.e., role modelling conversations) to support staff to become confident in having cancer care discussions with caregivers.
Improve support for family caregivers	
1. Help to reduce emotional and physical burden on informal caregivers.	Signpost to dementia and comorbid cancer‐specific support resources, e.g., macmillan and Alzheimer's society
Healthcare service‐level recommendations	
Improving connections with oncology services	
1. Creating informed ways of communication between oncology services and nursing homes settings	Implement the use of telehealth into nursing home and oncology care practice, to help provide easier and ‘minimally disruptive’ access to secondary service advice and support.

We found that ‘clinical cancer diagnoses’ (i.e. cancer is suspected but not confirmed) were not well‐recorded in residents' care documentation, certainly versus information about dementia and its symptoms. This contrasts with previous research in oncology secondary care that had variable documenting of dementia in medical records [23]. Without formal information and knowledge of a resident's cancer diagnosis, cancer‐related symptoms may be more likely to be overlooked or, through diagnostic overshadowing, misattributed to preexisting dementia, thus impeding effective symptom management. We suggest there was also a prioritisation of dementia‐focussed needs driving practice further risking inadequate attending to cancer‐related symptoms and needs [30, 31]. This offers a contributory explanation for why people with DCC are at a higher risk of unmanaged pain, increased emergency department admissions and hospitalisations, receive a cancer diagnosis post‐mortem and have a poorer quality of life [2, 3, 4, 24]. Therefore, implementation of strategies to promote assessment and continuous monitoring of cancer‐related symptoms is essential to deliver PCC.

We found residents with DCC were dependant on NHs to deliver effective symptom management for this resident group. Staff are with residents 24‐h a day and are crucial for the identification of and changes to symptoms [32]. However, in conjunction with limited knowledge and documentation of cancer‐related care needs, staff may be disempowered due to education and training for delivering symptom management and palliative care. Previous literature has identified key issues for NH staff included symptom management, unanticipated deterioration for decision‐making and avoiding advanced care planning conversations [33]. Thus, to improve the delivery of effective symptom management and palliative care for people with DCC, further training and support from specialist palliative care teams is required for NH staff.

### 4.1 Study Limitations

Data were collected from multiple NHs, though all located in Northern England, thus there may be limitations on the transferability of the findings and recommendations to other UK regions and internationally. Ethnography can be criticised due to its lack of objectivity and the influence of the researcher on the data [34]. As previously noted, the first author proactively minimised their lens of biases, for example, through keeping and reviewing a reflexive journal including their views and judgements [35], and regularly sharing and critically discussing their observation notes and ideas about theme development with the wider research and lay advisory teams. Due to limited resources, interviews with GPs were not possible.

### 4.2 Clinical and Research Implications

Based on our findings, we have outlined recommendations that may support NH staff to deliver PCC to residents with DCC (Table 2). Some of the proposed recommendations may require long‐term planning and careful consideration of resources and implementation or may be considered unfeasible due to time constraints and organisational challenges.

This study's findings and previous research shows DCC care requires complex and subtle judgements from HCPs when making referrals, but further research is required to understand the role of primary care in supporting people with DCC. Primary care HCPs (i.e. GPs) continue to support symptom management and delivery of palliative care if residents are unable to access hospital‐based support. Thus, we are conducting a National Institute for Health and Care Research funded study to identify ways to optimise cancer recognition, referral and management for people with dementia in primary and community care [36].

## 5 Conclusion

This study is the first to provide an overview and highlight the care disparities of a somewhat hidden resident group with DCC in NHs where cancer referral is deemed neither feasible nor appropriate. Implementing earlier conversations about feasible care outcomes, including hospital referral or care through palliation is crucial. Without appropriate recognition of a clinical‐only cancer diagnosis and support for NH staff it could lead to advancement of symptoms that might be challenging (i.e. pain) and less well managed. We outline several recommendations to support NH staff to deliver PCC to residents with DCC.

## Conflicts of Interest

The authors declare that the research was conducted in the absence of any commercial or financial relationships that could be construed as a potential conflict of interest.

## Supporting information

Supporting Information S1

## Data Availability

Research data are not shared.
