# Supplementary material for: Understanding Cancer Care for Nursing Home Residents Living With Dementia: An Ethnographic Study
Source: Psychooncology. 2025 May 31;34(6):e70184. doi: 10.1002/pon.70184 (PMC12126173; doi:10.1002/pon.70184)
Supplement: Supplementary file 1 — Supporting Information S1 [file PON-34-e70184-s001.docx]

**Observation Guide**

**General observations:**

- Description of nursing home settings (environment; layout; furniture & equipment; use of space; sight of others; sounds)
- Organisation (staffing; skill mix; time and workload pressures)
- Description of residents, families/supporters & staff (characteristics; roles; aims)
- Patterns of activity (daily routines; key events; movement through the space; timing, location & duration of events)
- Patterns of interaction (Who talks to who? About what? When? What are they trying to achieve?)
- Any differences in the above that occur as a result of, or in response to, memory problems.

**Individual resident observations:**

- Participants (Who? What is their background story?)
- Actions & interactions between residents with dementia and cancer (RWDC), caregivers/visitors & staff (content of actions/interactions, patterns, non-verbal communication, what does not happen)
- Experiences of RWDC within the home
- Involvement of RWDC and their informal caregivers (e.g. in treatment, care, decision making)
- Challenges, solutions and good practices when providing or treatment & care alongside memory problems (e.g. decision making; treatment choices, intervention priorities)
- Impacts of problems or good practices in providing cancer care for RWDC.

**Additional data sources:**

Information leaflets, posters and care plans.
